# Data-Driven Classification of Human Movements in Virtual Reality–Based Serious Games: Preclinical Rehabilitation Study in Citizen Science

**DOI:** 10.2196/27597

**Published:** 2022-02-10

**Authors:** Roni Barak Ventura, Kora Stewart Hughes, Oded Nov, Preeti Raghavan, Manuel Ruiz Marín, Maurizio Porfiri

**Affiliations:** 1 Department of Mechanical and Aerospace Engineering New York University Tandon School of Engineering Brooklyn, NY United States; 2 Department of Technology Management and Innovation New York University Tandon School of Engineering Brooklyn, NY United States; 3 Department of Physical Medicine and Rehabilitation Johns Hopkins School of Medicine Baltimore, MD United States; 4 Department of Quantitative Methods, Law and Modern Languages Technical University of Cartagena Cartagena Spain; 5 Murcia Bio-Health Institute (IMIB-Arrixaca) Health Science Campus Cartagena Spain; 6 Center for Urban Science and Progress New York University Brooklyn, NY United States; 7 Department of Biomedical Engineering New York University Tandon School of Engineering Brooklyn, NY United States

**Keywords:** motion analysis, principal component analysis, telerehabilitation, virtual reality

## Abstract

**Background:**

Sustained engagement is essential for the success of telerehabilitation programs. However, patients’ lack of motivation and adherence could undermine these goals. To overcome this challenge, physical exercises have often been gamified. Building on the advantages of serious games, we propose a citizen science–based approach in which patients perform scientific tasks by using interactive interfaces and help advance scientific causes of their choice. This approach capitalizes on human intellect and benevolence while promoting learning. To further enhance engagement, we propose performing citizen science activities in immersive media, such as virtual reality (VR).

**Objective:**

This study aims to present a novel methodology to facilitate the remote identification and classification of human movements for the automatic assessment of motor performance in telerehabilitation. The data-driven approach is presented in the context of a citizen science software dedicated to bimanual training in VR. Specifically, users interact with the interface and make contributions to an environmental citizen science project while moving both arms in concert.

**Methods:**

In all, 9 healthy individuals interacted with the citizen science software by using a commercial VR gaming device. The software included a calibration phase to evaluate the users’ range of motion along the 3 anatomical planes of motion and to adapt the sensitivity of the software’s response to their movements. During calibration, the time series of the users’ movements were recorded by the sensors embedded in the device. We performed principal component analysis to identify salient features of movements and then applied a bagged trees ensemble classifier to classify the movements.

**Results:**

The classification achieved high performance, reaching 99.9% accuracy. Among the movements, elbow flexion was the most accurately classified movement (99.2%), and horizontal shoulder abduction to the right side of the body was the most misclassified movement (98.8%).

**Conclusions:**

Coordinated bimanual movements in VR can be classified with high accuracy. Our findings lay the foundation for the development of motion analysis algorithms in VR-mediated telerehabilitation.

## Introduction

### Stroke Telerehabilitation

Stroke is continuously cited as a leading cause of disability in adults. Every year, 795,000 Americans experience stroke, and 649,000 survive it [1]. Approximately 610,000 of these cases are the first attacks, indicating that the population of stroke survivors is rapidly increasing [1]. Stroke survivors commonly experience neuromuscular disorders that profoundly disrupt their lives. It is estimated that 74% of stroke survivors require assistance with activities of daily living, costing billions of dollars annually [1,2]. Beyond loss of mobility, stroke-induced disability takes a societal toll; many stroke survivors can no longer contribute to the workforce and lose their functional role in their community [2]. They often enter a downward spiral that is associated with a steep decline in their psychological and cognitive well-being, affecting their families and social circles [1,3,4].

Motivated by these economic and societal needs, rehabilitation medicine aims to reintegrate individuals with disabilities into society. This process typically involves multiple visits to outpatient clinics, where therapists treat patients with arduous exercises. The more frequently and intensely they exercise, the sooner the patients would recover muscle strength and function [5]. Nonetheless, outpatient clinics are often underequipped and understaffed. As a result, patients have to wait for long periods for appointments and do not receive sufficient care, significantly hindering their recovery [6]. To address this issue, the notion of telerehabilitation has emerged.

In the ideal telerehabilitation paradigm, patients are prescribed home-based exercises involving electronic devices that measure their movements [7-9]. Data on motion are then sent to a physician, who would, in turn, remotely assess motor performance and recommend the next steps in the rehabilitation regimen. Through this process, patients are expected to exercise at their own convenience at home, readily receive professional feedback, and ultimately maximize their rehabilitation outcomes. Multiple telerehabilitation systems have been introduced in the past 20 years, demonstrating and yielding outcomes comparable with those of traditional in-clinic rehabilitation [9-12].

Despite the promising prospects, the advantages of telerehabilitation are often not realized, as patients fail to adhere to their prescribed regimen in the absence of a physical therapist [13,14]. One of the primary factors pinpointing a lack of adherence is a lack of motivation [13,14]. To address this critical limitation, innumerable efforts were invested in gamification of telerehabilitation [15-17]. Notably, the Java Therapy, one of the first examples of a telerehabilitation system, incorporated *therapy games* in between *status tests* that measure rehabilitation progress [18]. Similarly, games that involve chasing rabbits [19], catching falling fruit [20], and even competitive air hockey [21], were developed to make physical exercise more enjoyable.

### Citizen Science–Based Telerehabilitation

Although games effectively improve engagement in telerehabilitation, incorporating citizen science into the activity was proposed instead [22]. In citizen science, members of the general public carry out research tasks in projects led by professional scientists [23,24]. These tasks involve data collection or data analysis and do not require any particular expertise or commitment [23,24]. Citizen science is a compelling means for improving engagement in telerehabilitation for a few reasons. Similar to games, the motivations underlying participation in citizen science are primarily intrinsic [25,26]. Some citizen science projects incorporate gaming elements, such as point systems, scoreboards, or competitions, to promote long-term participation [27,28]. Unlike in games, citizen scientists choose to contribute to a project not only because it is enjoyable or fun but also because they are interested in the research topic, they have a desire to learn more about it, and they would like to promote it [29-31]. In essence, citizen science is intellectually stimulating and encourages learning. Moreover, citizen science has the potential to empower patients to help scientists despite their disability, increase their self-esteem, and provide them with a sense of belonging to a community [24,32]. Finally, as it is important for leading scientists to collect or analyze data meticulously, there is rarely a time constraint for making a contribution such that users can contribute at their own pace.

In a recent study, we presented a low-cost telerehabilitation system that delivers exercise in the context of citizen science [33]. The system consisted of a Microsoft Kinect sensor and an inertial measurement unit mounted on a wooden dowel. Users would manipulate the dowel in front of the Kinect sensor to perform actions on a standard computer monitor or television screen. More specifically, the actions involved the annotation of 360° images of a highly polluted canal in Brooklyn, New York, United States. The system was dedicated to bimanual exercise, in which users would manipulate the dowel with both hands. The system also featured a classification algorithm that identified the movements performed by the user, which achieved a high accuracy of 93.1%.

In this study, we adapted the Kinect-based interface to virtual reality (VR) and focused on the classification of upper limb movements in a preclinical setting. We recorded the interactions of 9 healthy users with the Oculus Rift (Oculus VR), a popular VR gaming system. The Oculus Rift consists of a head-mounted display, 2 Touch controllers, and 2 tracking sensors. Inertial measurement units are embedded in the head-mounted display and Touch controllers such that the system is able to record the orientation of the head and the hands. The devices were also seeded with an array of infrared lights, which, in conjunction with the tracking sensors, enable high-fidelity motion tracking through the Oculus trademarked Constellation Tracking [34]. The VR setting offered more degrees of freedom in motion relative to our Kinect-based system, whereby users could rotate their entire bodies to interact with the interface. Therefore, to adapt the software and classification algorithm, we applied a kinematic framework that infers the position and orientation of the Touch controllers relative to the head-mounted display.

Our choice to explore human movement in VR was motivated by 2 main reasons. First, the major barriers that prevent the widespread adoption of rehabilitation technologies are cost and user friendliness. Rehabilitation devices are often custom-made, cost prohibitive, and require technological proficiency that extends beyond the typical knowledge of the general public [35]. On the other hand, gaming controllers such as the Oculus Rift are safe and intuitive to use and are more affordable than rehabilitation robots, thereby offering a viable means for home-based telerehabilitation. Gaming controllers can also objectively measure the motor performance through their embedded sensors. Specifically, the Oculus Rift tracks movements of the headset and Touch controllers with high spatial and temporal resolution, thereby providing rich data on the user’s motions. It was validated in controlled experiments and deemed sufficient for motion analysis in medical applications [36,37].

Second, VR is the most immersive medium available today. The technological apparatus of VR grants the user the experience of *presence*, where the user accesses a novel environment and interacts with it as if the computer ceased to exist [38,39]. In the context of rehabilitation, immersive VR environments are largely used to improve patients’ engagement and adherence to the rehabilitation regimen, which will accelerate their recovery in return [6,16,40]. The literature suggests that patients undergoing rehabilitation augmented with VR could substantially improve their motivation and motor functions [40,41]. For example, Dockx et al [42] compared 281 older adults’ perceptions of fall prevention training over a period of 6 weeks when delivered with and without VR. All participants who exercised in the VR condition reported higher engagement and perceived benefits and were more likely to recommend the intervention to others than those who did not use VR in their training. In another study, AlMousa et al [43] tested a game with 5 patients with stroke and compared their satisfaction when playing in VR and in a traditional setting. All patients agreed that the VR modality was highly motivating and expressed interest in including it in their rehabilitation. Finally, in a study involving 4 patients with spinal cord injury, Palaniappan and Duerstock [44] showed that VR improved motor performance, whereby patients’ upper limb range of motion was greater.

**Figure 1:**
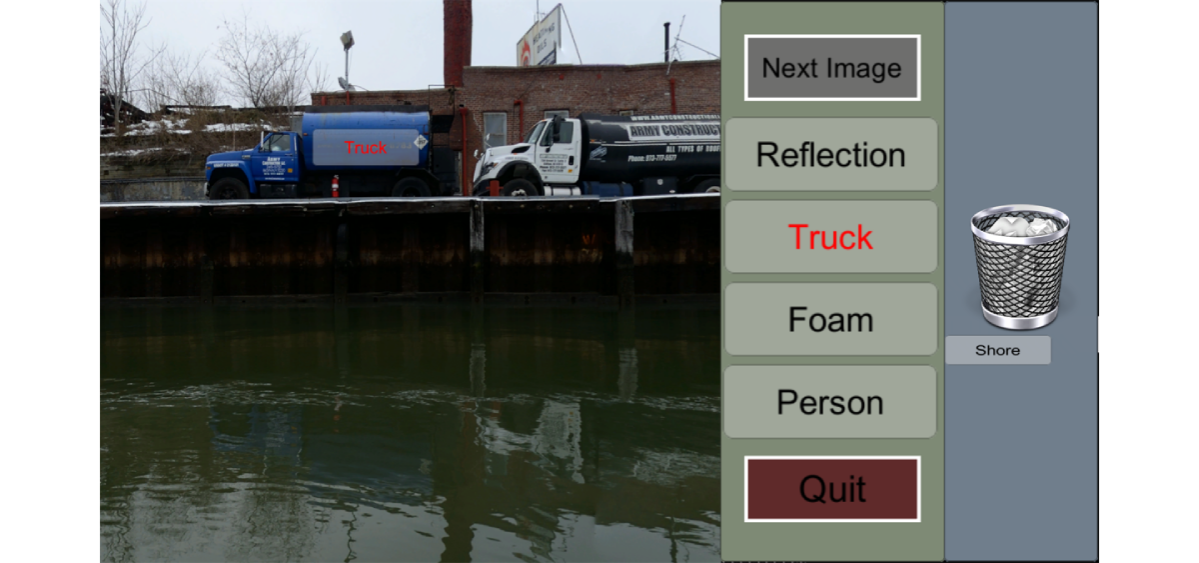
Screenshot of the user interface. A 360° image of a polluted canal can be explored in the virtual environment. In the green panel on the right, there is a list of 4 labels (*Reflection*, *Truck*, *Foam*, and *Person*) and a trash bin. The *Next Image* button above the labels allows the user to analyze a new image. Below the list, a *Quit* button is situated. By pressing it, the user will exit the application. The user has selected the label *Truck* (highlighted in red) and intends to allocate it onto the image. The label *Shore* has been disposed of and appears below the trash bin.

We created an interactive interface in which users could participate in an environmental citizen science project. In this particular application, users contributed to the environmental monitoring of the highly polluted Gowanus Canal in Brooklyn, New York, United States. Users could explore 360° images of the canal, select labels from a list of 4 labels, and allocate them onto objects of interest, such as potential pollutants and notable landmarks (Figure 1).

The interface was dedicated to bimanual training of patients with stroke, whereby users interacted with the interface by performing coordinated movements with both arms. Many rehabilitation strategies, such as constraint-induced movement therapy [45,46], task-oriented training [47], and continuous passive movement [48], have various advantages. Bimanual training is highlighted as a potent clinical approach for the recovery of coordinated movements with both physiological and practical advantages [49] Research has shown that passive movement of paretic limbs can recover voluntary motion by imparting electrical impulses to the contralateral primary motor cortex (sometimes referred to as *spillover*) [50-52] and project them to the affected muscles [53-55]. Furthermore, it has been argued that bimanual skills are abundant in activities of daily living and therefore practicing them will help patients regain independence more quickly [56-59].

We pursued a simple, yet effective, data-driven approach to automatically assess bimanual movements in VR.

### Motor Assessment Using Machine Learning

Machine learning offers an important avenue for automatically identifying and categorizing human behavior. In machine learning, a computer uses data to predict an outcome without explicitly knowing the relationship between the data and the outcome [60,61]. The input of a machine learning algorithm consists of features that describe instances of data. When a supervised machine learning approach is used, knowledge of the outcome must be available during training. In this case, a set of instances is fed to the machine, encapsulating their features and associated outcomes [61,62]. For example, Begg and Kamuzzaman [63] used machine learning to distinguish between the gait of the young and older adults. The authors fed their machine learning algorithm with data on the gait of 12 young and 12 older individuals, where their gait was summarized through multiple features, such as stride length, walking speed, forces applied by the feet, and ankle angles. They used a supervised machine learning approach (support vector machine [SVM]) and therefore provided the machine with the true class of the participant: young or older. Following training, the SVM classifier achieved an accuracy rate of 91.7% in the classification of the age group of the participant.

In a similar study, Novak et al [64] aimed to identify gait initiation and termination using wearable inertial measurement units. The authors recorded 10 participants walking with inertial sensors on their legs and trained a tree classifier to distinguish between gait phases. The algorithm exceeded 80% accuracy and was robust with respect to the gait speed. Semwal et al [65] trained a multilayer perceptron to identify disordered gait. The authors defined features for walking, running, jogging, and jumping from vision-based and sensor-based data and achieved accuracy rates ranging from 85% to 92.5%.

Despite its success with gait analysis, the use of machine learning to assess upper limb movement has not been extensively studied. Such an assessment is more challenging as the repertoire of arm movements is wider than that of the lower limbs. In several studies, statistical pattern recognition algorithms have been used to quantify the motor performance of the upper limb from data collected by inertial sensors [66] and vision-based sensors [67]. Additional work to recognize upper limb movement was carried out using k-means clustering and convolutional neural networks [68,69]. Nonetheless, the efficacy of machine learning in upper limb rehabilitation remains underexplored.

### Objective

We developed a machine learning algorithm that classifies the movements performed by the user to automate the assessment of motor performance. The proposed algorithm implements dimensionality reduction through principal component analysis (PCA), feature extraction, and ensemble classification. In all, 9 healthy individuals interacted with our interface, whereas data on their movements were recorded by the sensors embedded in the Oculus Rift devices. The classification of the movement was achieved with remarkably high accuracy and could reduce the time and cost of poststroke rehabilitation assessment by a therapist. Furthermore, the classification strategy can be extended to provide haptic feedback to the user to perform exercises correctly and safely.

## Methods

### VR Interface

The interface was developed in the Unity real-time game engine (Unity Technologies) for use with the Oculus Rift VR system. In the game, participants were presented with a random 360° image of the Gowanus Canal, overlaid by a heads-up display (HUD). The HUD served as the participants’ main method of interacting with the application. It contained a button for navigation between images of the canal and a trash bin and a list of descriptive keywords that may or may not describe objects within the image.

Users were tasked with analyzing the images. Specifically, they could explore the 360° images, select labels from the list of keywords, and allocate them to objects of interest (Figure 1). If the users could not find an object that a label described in the image, they could eliminate the label by allocating it onto the trash bin (Figure 1). Once the user felt that the image was saturated with labels, they could analyze a new image by selecting the *Next Image* button.

**Figure 2:**
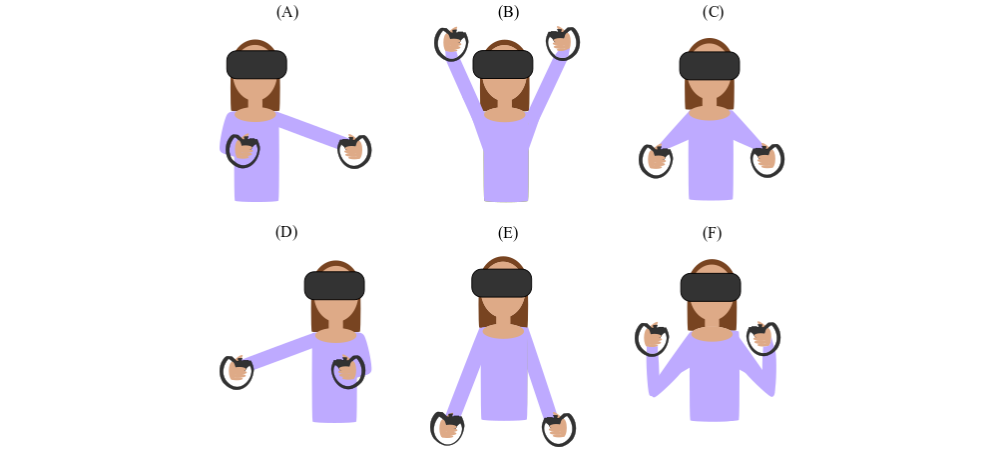
Implementation of the user interface. The user is able to perform actions on a computer through (A and D) horizontal abduction and adduction of the shoulders, (B and E) flexion and extension of the shoulders, and (C and F) flexion and extension of the elbows.

To interact with the HUD, the users performed bimanual gestures (Figure 2). Specifically, users began from a baseline pose where they flexed their elbows and held the Touch controllers near their shoulders. To move the cursor to the left, they extended both arms to the left side of their body, simultaneously performing horizontal abduction of the left shoulder, horizontal adduction of the right shoulder, shoulder flexion, elbow extension, and forearm pronation (Figure 2A). Similarly, to move the cursor to the right, they performed horizontal shoulder abduction in the opposite direction, extending both hands to the right side of their bodies (Figure 2D). To move the cursor upward, users raised the Touch controllers by flexing their shoulders and extending their elbows (Figure 2B). To move the cursor downward, they extended both the elbows and lowered the Touch controllers (Figure 2E). Finally, to select a button, they flexed both shoulders simultaneously and extended their elbows, pushing the Touch controllers away from their body (Figure 2C and Figure 2F). These movements used most joints of the upper limb and were commonly prescribed to patients [70,71]. If a user wanted to move the cursor diagonally along the screen, they would instead move it horizontally and vertically.

**Figure 3:**
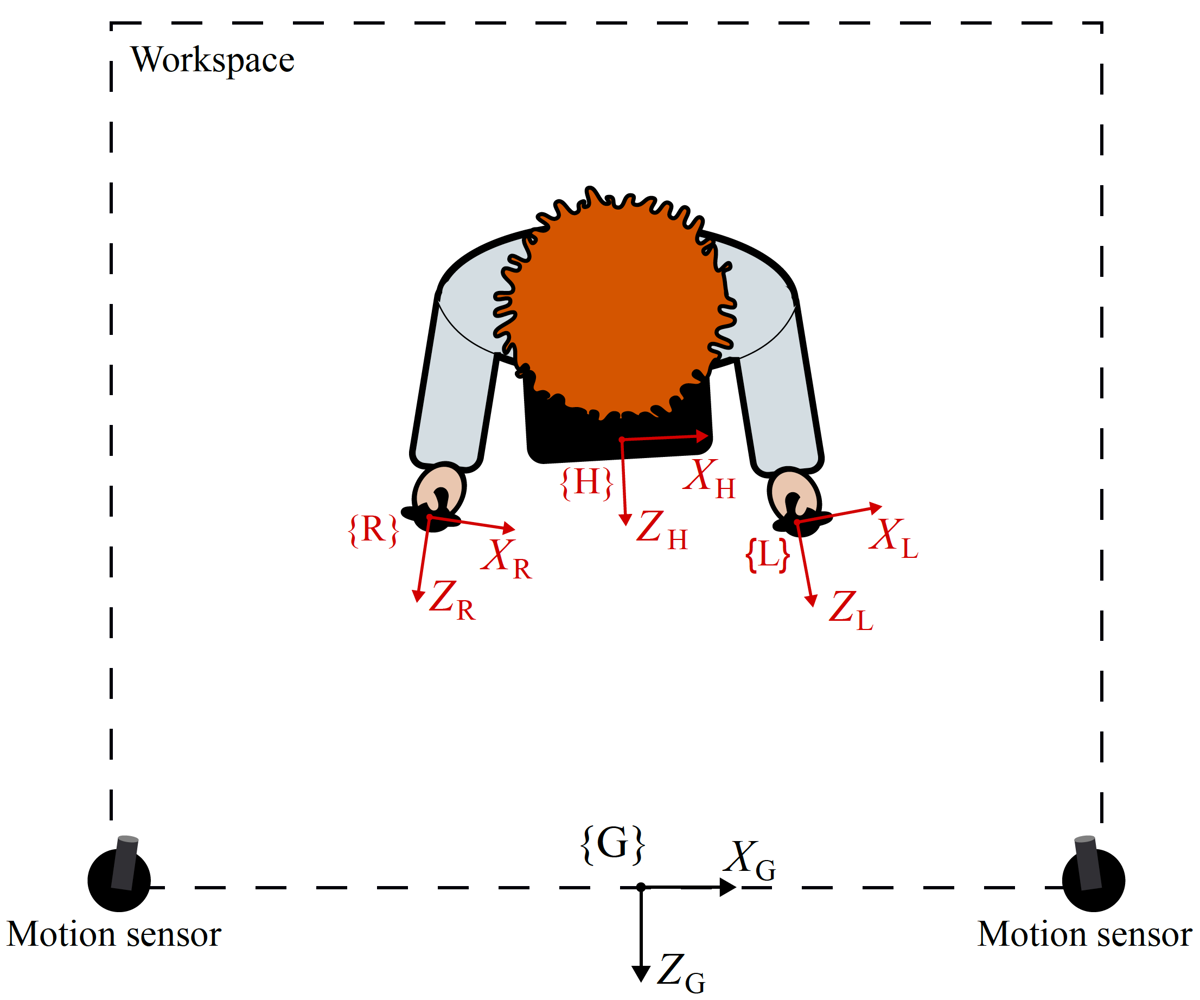
Illustration of a typical Oculus Rift workspace from a top view. Two sensors are placed at the edge of the workspace. The global frame, {G}, uses the coordinate system (*X*_G_, *Y*_G_, *Z*_G_). The local frames for the head-mounted display and the right and left Touch controllers are drawn in red and denoted as {H}, {R}, and {L}, respectively.

To enable the user interface, we used a kinematic framework using data on the position of the head-mounted display and Touch controllers, measured by the infrared camera sensors. We considered 4 reference frames for the inertial, global space, denoted as {G} and the 3 noninertial reference frames associated with the head-mounted display, right hand Touch controller, and left hand Touch controller, denoted as {H}, {R}, and {L}, respectively (Figure 3).

**Figure 4:**
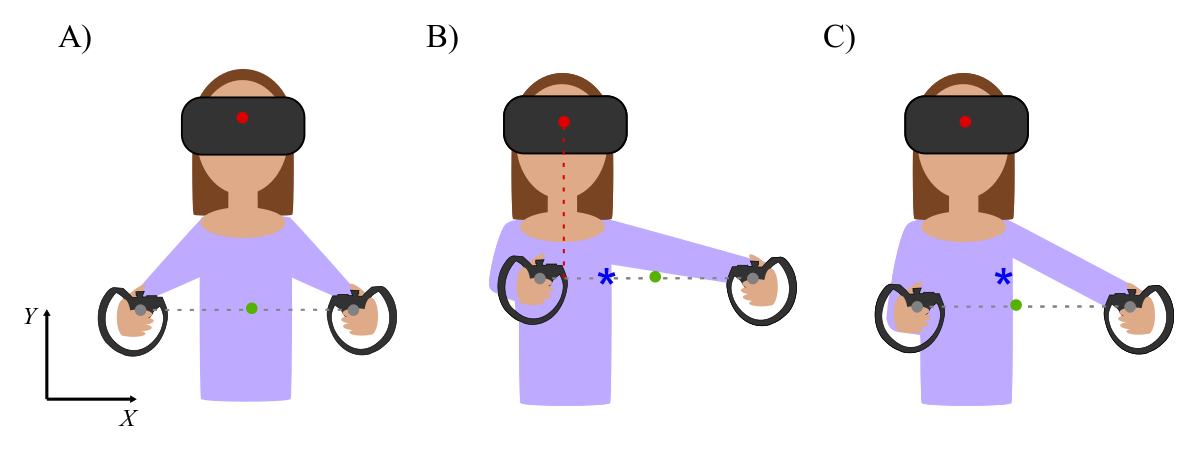
Illustration of the calibration threshold along the *X*-axis. (A) Throughout the game, the instantaneous position of the point between the Touch controllers (marked with a green circle) is computed. (B) Its maximum position relative to the position of the head-mounted display along the *X*-axis (marked with a red circle) is computed during the calibration phase. A threshold is set at 25% of that displacement, represented by the blue star. (C) During the game, every time the average controller point exceeds the threshold, the cursor will begin moving on the screen in the corresponding direction.

Throughout the game, a midway point between the Touch controllers, *P*^G^_h_ (Figure 4A), was computed in real time as follows:



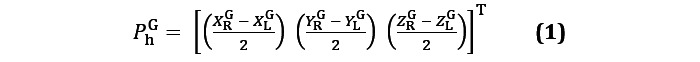



where *P*^G^_h_ is a vector in the form of [*X Y Z*]^T^ that expresses the position of the midpoint *h* in the global frame {G} (T being matrix transposition); 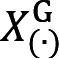, 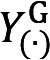, and 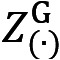 are the positions of a point along the *X*-, *Y*-, and *Z*-axis of global frame {G}, respectively; and subscripts R and L represent the right and left Touch controllers, respectively. The cursor on the screen responded to the fixed values of *P*^G^_h_. For example, if *X*^G^_h_ was greater than a certain threshold value, the cursor would move to the left on the screen. Similarly, if *X*^G^_h_ was smaller than a certain negative threshold value, the cursor would move to the right. Considering that patients may take longer to complete their movements, we did not impose any time constraints on these controls.

To accommodate for impaired movement with a compromised range of motion, a calibration phase was added to determine the aforementioned threshold values. During calibration, the participant performed each of the movements 5 times consecutively. The software computed an average of the user’s range of motion 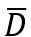 during the 5 iterations as follows:



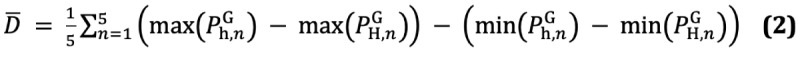



where *n*=1, 2,..., 5 is the iteration of the movement, *P*^G^_h,*n*_ is the time series of the position of the midpoint between the right and left Touch controllers during iteration *n*; and *P*^G^_H,*n*_ is the time series of the position of the head-mounted display during iteration *n*. The application set a threshold point at a distance of 0.25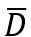 along the *X*-, *Y*-, and *Z*-axis of the head-mounted display (Figure 4B). At any time when *P*^G^_h_ exceeded 0.25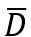, the cursor began moving on the screen along the axes that satisfied this condition (Figure 4C). Thus, users who had a limited range of motion had to move their arms at a shorter distance to induce movement of the cursor on the screen.

Finally, acknowledging that physical therapy can be physically and mentally taxing, we enabled a *Home* page menu such that patients could press a button to pause the software and rest. This feature is particularly important for telerehabilitation of stroke, as many patients may feel pain or fatigue, discouraging them from engaging in the exercise [14].

### Data Collection

This study was carried out in accordance with the relevant guidelines and regulations set by the New York University’s Institutional Review Board, the University Committee on Activities Involving Human Subjects (study number: FY2019-2828). Informed consent for participation was obtained from all participants.

In all, 9 members of the university community were recruited and escorted to a private room. They were introduced to the project and VR system. Upon signing a consent form, the participants stood in a 3 meter × 3 meter cleared space and wore the head-mounted display. They viewed a short presentation about the Gowanus Canal and the notion of citizen science and underwent a calibration phase.

The calibration was designed such that the participants began with a baseline pose with their elbows bent and hands held near their respective shoulders. The participants first performed horizontal shoulder abduction toward their right side. Instructions on the screen explicitly asked the participants to extend their arms as far as possible to the right and return to the baseline pose, repeating this movement 5 times. Then, the participants performed horizontal shoulder abduction toward their left side and returned to the baseline pose 5 times. In the same manner, the participants performed shoulder flexion by raising both hands, elbow extension by lowering both hands, and simultaneous shoulder flexion and elbow extension by pushing both hands forward in this order. The participants repeated each movement 5 times consecutively and returned to the baseline pose after each excursion.

After calibration, the participants completed a tutorial teaching them how to use the HUD. They then analyzed images of the Gowanus Canal for as long as they wished. The movements of the participants were recorded throughout the experiment. The data set consisted of the time series of the positions of the head-mounted display and Touch controllers in 3D and their orientations in Tait-Bryan angles. Measurements were logged at a sampling rate of 89 measurements per second.

### Data Analysis

#### Kinematics in the VR Setting

Data were processed and analyzed in MATLAB (MATLAB R2020a; The MathWorks, Inc). We aimed to infer the participants’ movement during their interaction with the VR system from data on the positions and orientations of the head-mounted display and Touch controllers. In VR, the interface is not constrained to a fixed planar screen, and participants’ interactions extend to 3D whereby the user can walk and turn their body around. Therefore, to infer the participants’ movements, the positions of their hands relative to their heads are more informative than their positions in absolute space.

We began with a kinematic description of the positions and orientations of the Touch controllers relative to the head-mounted display through matrix manipulation [72]. The reference frame of the head-mounted display was expressed with respect to the global frame using the rotation matrix:



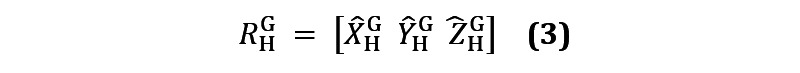



where a superimposed hat identifies unit vectors for the reference frames, such that the columns of the matrix are the unit vectors of {H}, expressed in {G}’s coordinate system. Similarly, the reference frames of the Touch controllers with respect to the global frame were expressed as *R*^G^_R_ and *R*^G^_L_ for the right and left controllers, respectively.

Taking the devices’ rotation matrices, the frame of reference of the right Touch controller relative to the head-mounted display was calculated as



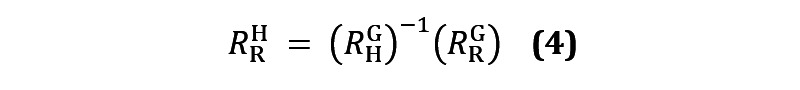



and the left Touch controller’s was calculated as



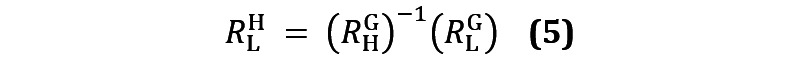



where the inverse is equivalent to the transpose of the matrix [72]. To fully describe the instantaneous relative positions and relative orientations of the devices, we applied the homogeneous transform [72] at each time step, such that



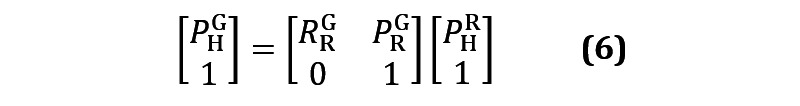



where *P*^G^_H_ is the instantaneous position of the head-mounted display in the global frame, *P*^G^_H_ is the instantaneous position of the head-mounted display in the right hand controller frame, *R*^G^_R_ is the rotation matrix of the right Touch controller frame relative to the global frame, *P*^G^_R_ is the position of the right Touch controller in the global frame, and 0 on the bottom left entry of the matrix represents a row vector of 3 zeros. We applied the transformation to instantaneous measurements at each time step and generated a time series containing the positions and orientations of the Touch controllers relative to the head-mounted display.

#### Assessing Motor Performance

For a comparison of patients’ movements with movements of healthy ones, we quantified the participants’ motor performance using several metrics: (1) range of motion, computed as the maximum distance of each of the Touch controllers from the headset, along each of the anatomical planes [22,73]; (2) mean speed, computed as the average of instantaneous speeds [22,73]; (3) smoothness, computed as the mean speed divided by the maximal instantaneous speed [22,73]; and (4) path length, measured as the sum of distances between pairs of consecutive data points during movement [22,74].

#### Feature Selection

We pursued a data-driven methodology to classify the movements performed by the participant based on the Touch controllers’ position and orientation relative to the head-mounted display. Only data from the calibration phase were used in the analysis, as the sequence of movements performed by the participants during this period was known and could be specified in supervised training. The data that were collected in the remainder of the session while participants interacted with the citizen science software could be used in future endeavors to assess motor performance and engagement over longer periods, once automatic classification is implemented. We also included the instantaneous head-mounted display and Touch controllers’ linear and angular velocities in the global frame for analysis. Specifically, we computed the devices’ linear, denoted as 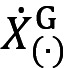, 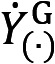, and 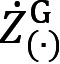, where (∙) is the noninertial reference frame under examination and angular velocities about their *X*-, *Y*-, and *Z*-axis in the global reference system, denoted as 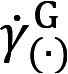, 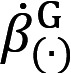, and 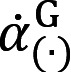. We also computed the Touch controllers’ positions and orientations relative to the head-mounted display, denoted as 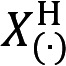, 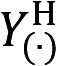, and 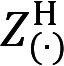, and 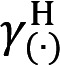, 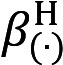, and 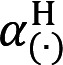, respectively. In general, we denoted 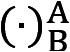 as the generic coordinate of point B, in coordinate system {A}. For notational convenience, when the trailing subscript is a reference frame, B represents the position of the origin of frame {B}. For example, *X*^H^_R_ is the position of the right Touch controller, along the *X*-axis of the head-mounted display. Similarly, *γ*^G^_R_ is the angular velocity of the right Touch controller about the *X*-axis in the global frame. Overall, the data set included 30 variables, as summarized in Table 1.

**Table 1:** Summary of the variables used for principal component analysis. The variables *γ*, *β*, and *α* refer to the Tait-Bryan angles of the Oculus head-mounted display and Touch controllers about the *X*-, *Y*-, and *Z*-axis, respectively.

Device and variable notation			Variable description
**Head-mounted display**			
	*X*^G^_H_, *Y*^G^_H_, *Z*^G^_H_	Linear velocity in {G}	
	*γ*^G^_H_, *β*^G^_H_, *α*^G^_H_	Angular velocity in{G}	
**Right Touch controller**			
	*X*^H^_R_, *Y*^H^_R_, *Z*^H^_R_	Position in {H}	
	*γ*^H^_R_, *β*^H^_R_, *α*^H^_R_	Orientation in {H}	
	*X*^G^_R_, *Y*^G^_R_, *Z*^G^_R_	Linear velocity in {G}	
	*γ*^G^_R_, *β*^G^_R_, *α*^G^_R_	Angular velocity in {G}	
**Left Touch controller**			
	*X*^H^_L_, *Y*^H^_L_, *Z*^H^_L_	Position in {H}	
	*γ*^H^_L, _*β*^H^_L_, *α*^H^_L_	Orientation in {H}	
	*X*^G^_L_, *Y*^G^_L_, *Z*^G^_L_	Linear velocity in {G}	
	*γ*^G^_L, _*β*^G^_L_, *α*^G^_L_	Angular velocity in {G}	

Next, we automatically identified instances of movement (versus nonmovement) in the time series of each variable and segmented them. Specifically, we used finite differences between the positional data for the Touch controllers with respect to time and defined the time series [75]:



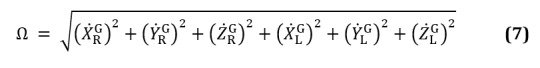



**Figure 5:**
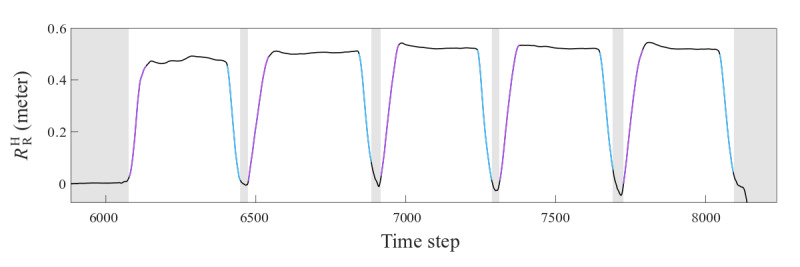
Example of movement segmentation. The time series reflects the first 5 movements a participant performed during the calibration phase. The colored intervals are the ones identified as instances of movement in the segmentation process. Purple intervals correspond to outward movements where the participant extended their arms, and blue intervals reflect subsequent abduction when the participant returned to baseline pose. Gray regions are segments where the participant assumed the baseline pose.

Intervals of movement were taken as the instances where *Ω* exceeded 0.077 meters/second and lasted for longer than 0.2 seconds (Figure 5). These threshold values were derived empirically and were unique to the participant. To identify instances where a distinct pose occurred, pairs of consecutive intervals and the time series between them were selected as segments. Overall, 25 segments were identified, one each for each movement.

PCA was performed to identify salient variables in each movement. Within segments *n*=1, 2,..., 25, each of the 30 time series was normalized with respect to its own SD in the segment. The normalized time series, *s*_*n*,*i*_ was represented by a column vector containing variable *i*=1, 2,..., 30 in segment *n*. For each segment *n*, we generate covariance matrix *K*_*n*_, whose entries *i*, *j* are given by



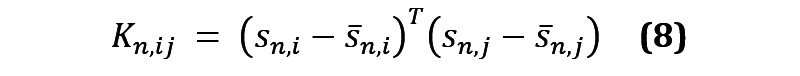



where *i*=1, 2,..., 30, *j*=1, 2,..., 30, and 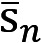 is the average value of the components of vector *s*_*n*_. As there are 30 variables, there are 30×30 possible ordered variable pairs to compute the covariance for, which is the size of the symmetrical matrix *K*_*n*_.

**Figure 6:**
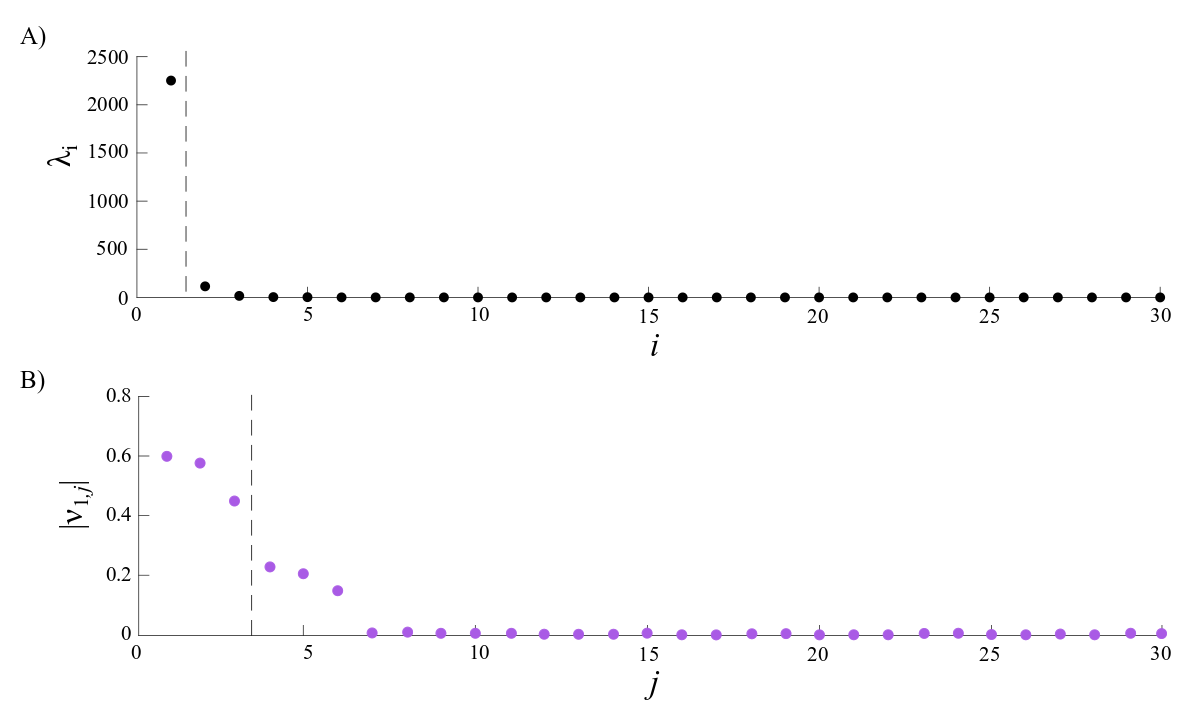
Example of the spectrum of a covariance matrix, corresponding to shoulder abduction to the right. The covariance matrix quantified the covariance of the 30 variables in the first segment, corresponding to shoulder abduction to the right side. (A) The array of 30 eigenvalues (*λ_i_s*) of the covariance matrix is sorted in descending order. The spectral gap where the largest difference between 2 consecutive eigenvalues appears (marked with a vertical dashed line) indicates that the eigenvector *v_i_*, which is associated with the largest *λ_i_*, is sufficient for capturing most of the variance in this first segment. (B) The absolute values of the components of *v_i_* are sorted in descending order as well to identify the principal components. Here, the largest difference appears after 3 components, suggesting that the 3 variables associated with the first 3 components (in this case, *γ*^H^_L_, *α*^H^_L_, *β*^H^_L_) are principal for variation in the segment.

The principal components of each covariance matrix *K*_*n*_ were determined from the dominant eigenvalues *λ_i_s* [76]. To identify these eigenvalues, we defined a spectral gap as the largest difference between consecutive eigenvalues sorted in descending order (Figure 6A). The eigenvalues that preceded the gap were deemed to be dominant. Then, we examined the contribution of eigenvector *v*_*i*_’s components, the so-called *principal component loadings*, to these principal components. We sorted the absolute values of these loadings in descending order and recognized a gap as the largest difference between consecutive values. The loadings that appeared before the gap were retained, and the associated variables were used as salient variables that summarize the entire principal component (Figure 6B).

The salient variables we identified in the PCA were used to create discriminating statistics for training a classification algorithm. In the training, given the true class of a movement that was performed, the algorithm would unveil different relationships between the features that distinguish one movement from another [61,62].

Importantly, we observed that only the orientations of the Touch controllers relative to the head-mounted display were prominent during movement. Thus, their means and SDs were selected as the features. We also included the mean positions of the Touch controllers relative to the head-mounted display as features to further support the distinction between the poses. Nonetheless, we acknowledged that movements may be better discriminated using features that encapsulate the interactions between the variables. Therefore, we used correlation coefficients as additional features that relate 2 variables at a time. The correlation coefficients between *γ*, *β*, and *α* of one Touch controller and their counterparts in the other Touch controller were added to the analysis, yielding 21 features in total.

#### Movement Classification

We implemented a supervised machine learning classification that identifies which movement a user performs at any given time. To observe the evolution of features over time in a future clinical study, we chose to perform classification in a moving-window paradigm. Within this paradigm, we evaluated the actual movement and associated features within a window of several time steps, shifted the window forward in time by a single step, evaluated the features again, and so on. The length of the moving window was set to 13 time steps, equivalent to 0.15 seconds.

First, we established the true classes within each frame to train the algorithm. We visually inspected the time series of the calibration (where we knew what movement was performed), identified which movement was performed (if any) at every time step, and labeled it as such. Beginning from the first time step, we determined the true class of the window that covered the subsequent 13 time steps based on their mode. That is, the window’s true class matched the class of the majority of time steps (7 or more). Henceforth, the window was moved to the following time step and the subsequent true class was determined. In this manner, we created a time series for the true class of frames. To determine the true class of a movement within a 13–time step frame, we also computed the set of 21 features and recorded them for the same frame. Thus, we created 21 additional time series, each representing the evolution of a feature.

Next, we trained a classification algorithm using MATLAB’s Classification Learner app. We compounded the moving frames’ true classes and features across participants into a single table and selected it as the data set variable. The frames’ true classes were set as response variables, and all features were set as predictors. We applied a K-fold cross-validation with *K*=5, such that 80% of the calibration data from all participants were used for training and the remaining 20%, for validation. Finally, we selected bagged trees as the model type.

Bagged trees is an ensemble method based on decision trees [77]. A basic decision tree splits the input data into subgroups with a similar response to a binary criterion. The subgroups are partitioned recursively until the model is able to predict the output based on the class that has the majority representation. A bagged trees classifier performs bootstrapping and aggregation, that is *bagging*, on a multitude of decision trees. Specifically, the bagged trees algorithm generates decision trees by resampling the data set with replacement and determines the response class based on the simple majority of the trees’ predictions. Thus, this classification method mitigates the high variance often observed in them [78,79].

Because the trees are produced by bagging, all features are considered for a splitting event. It is possible to score the importance of each feature by estimating the *out-of-bag* error. That is, instances that were not sampled when a tree was generated were used to make a prediction. The mean error of the prediction was then computed. The features that yielded the largest decrease in mean error were considered to be the most important.

## Results

### Data Collection

Data were collected from 9 healthy participants who interacted with the interface. On average, the participants interacted with the interface for 368.26 (SD 92.74) seconds, generating time series of 32,776 (SD 8254) time steps on average. A total of 294,983 measurements were collected, of which 142,916 time steps (1605.80 seconds) were recorded during the calibration phase.

### Motor Performance

The participants’ range of motion, mean speed, peak speed, and path length were computed (Table 2). The range of motion, mean speed, and smoothness for each movement in one arm were comparable with those of its symmetrical counterpart. However, during shoulder adduction and shoulder flexion or extension upward, considerable variation was measured among participants with respect to smoothness; SDs were >25% of the mean value, or even greater than the mean value, as in the case of the left hand during shoulder flexion or extension upward. Finally, in all movements, the path length was larger than the range of motion, indicating that the participants did not follow a straight line along the anatomical axes.

**Table 2:** A summary of participants’ motor performance for each arm, computed from data from the right and left Touch controllers. The values represent the mean (SD) across the participants.

Movement and hand			Range of motion (meters), mean (SD)		Speed (meters/second), mean (SD)		Smoothness, mean (SD)		Path length (meters), mean (SD)
**Shoulder adduction to the right**									
	Right	0.61 (0.11)		0.86 (0.25)		2.47 (1.16)		0.72 (0.17)	
	Left	0.39 (0.06)		0.60 (0.13)		1.95 (0.58)		0.48 (0.08)	
**Shoulder adduction to the left**									
	Right	0.38 (0.05)		0.60 (0.10)		2.27 (1.43)		0.46 (0.08)	
	Left	0.61 (0.16)		0.94 (0.25)		4.39 (3.96)		0.74 (0.18)	
**Shoulder flexion or extension upward**									
	Right	0.60 (0.08)		0.86 (0.20)		3.43 (3.33)		0.64 (0.10)	
	Left	0.59 (0.08)		0.86 (0.20)		3.16 (3.64)		0.63 (0.10)	
**Shoulder flexion or extension downward**									
	Right	0.66 (0.06)		1.02 (0.30)		1.94 (0.21)		0.82 (0.13)	
	Left	0.66 (0.06)		1.03 (0.29)		1.95 (0.21)		0.81 (0.12)	
**Elbow flexion or extension upward**									
	Right	0.45 (0.05)		0.79 (0.19)		1.81 (0.29)		0.51 (0.08)	
	Left	0.45 (0.05)		0.78 (0.18)		1.81 (0.33)		0.50 (0.07)	

### Dimensionality Reduction

PCA disclosed the salient variables that best characterized each movement performed by the participants. Examination of the spectra of the covariance matrices revealed that the spectral gap was located between the largest and second largest eigenvalues for all instances of movement. Therefore, only 1 principal component was required to capture variations in movements.

Unexpectedly, among the 30 variables we considered, only the orientations of the Touch controllers were pertinent for the analysis. We found that shoulder abduction to the right side of the body and to the left side of the body were both associated with changes in the Tait-Bryan angles about the *X*- and *Z*-axis of the Touch controllers in the head-mounted display frame: *γ*^H^_R_, *α*^H^_R_, *γ*^H^_L_, and *α*^H^_L_. Shoulder flexion while raising the hands was dominated by variations in all 6 Tait-Bryan angles *γ*^H^_R_, *β*^H^_R_, *α*^H^_R_, *γ*^H^_L_, *β*^H^_L_, and *α*^H^_L_. Only changes in *α*^H^_L_ and *γ*^H^_L_ strongly characterized elbow extension while lowering the Touch controllers. Finally, appreciable variations in *α*^H^_R_ and *α*^H^_L_ were most prominent during elbow extension while pushing the Touch controllers forward. Changes in *γ*^H^_L_, *β*^H^_L_, *γ*^H^_R_, and *β*^H^_R_ were also detected in this motion. The PCA results are summarized in Table 3.

**Table 3:** Summary of the principal component analysis results. The variables *γ*, *β*, and *α* refer to the Tait-Bryan angles of the Touch controllers about the *X*-, *Y*-, and *Z*- axis, respectively.

Movement	Salient variables
Shoulder abduction to the right	*γ*^H^_R_, *α*^H^_R_, *γ*^H^_L_, *α*^H^_L_
Shoulder abduction to the left	*γ*^H^_R_, *α*^H^_R_, *γ*^H^_L_, *α*^H^_L_
Shoulder flexion or extension upward	*γ*^H^_R_, *β*^H^_R_, *α*^H^_R_, *γ*^H^_L_, *β*^H^_L_, *α*^H^_L_
Shoulder flexion or extension downward	*γ*^H^_L_, *α*^H^_L_
Elbow flexion or extension upward	*γ*^H^_R_, *β*^H^_R_, *α*^H^_R_, *γ*^H^_L_, *β*^H^_L_, *α*^H^_L_

### Feature Selection

We created features based on the variables identified as salient using PCA. We considered the mean values and SDs of the Touch controllers’ Tait-Bryan angles. We also included the Touch controllers’ mean displacement relative to the head-mounted display to distinguish between static poses. We used correlation coefficients as additional features to capture the interactions between the variables. Specifically, we computed the correlation coefficients for the following three pairs: (*γ*^H^_R_, *γ*^H^_L_), (*β*^H^_R_, *β*^H^_L_), and (*α*^H^_R_, *α*^H^_L_). Overall, 21 features were selected (Table 4).

**Table 4:** Summary of the features and variables used in the training of the classification algorithm.

Features	Variables
Mean	*X*^H^_R_, *Y*^H^_R_, *Z*^H^_R_ *γ*^H^_R_, *β*^H^_R_, *α*^H^_R_ *X*^H^_L_, *Y*^H^_L_, *Z*^H^_L_ *γ*^H^_L_, *β*^H^_L_, *α*^H^_L_
SD	*X*^H^_R_, *Y*^H^_R_, *Z*^H^_R_ *γ*^H^_R_, *β*^H^_R_, *α*^H^_R_
Correlation coefficient	(*γ*^H^_R_, *γ*^H^_L_), (*β*^H^_R_, *β*^H^_L_), (*α*^H^_R_, *α*^H^_L_) *X*^G^_L_, *Y*^G^_L_, *Z*^G^_L_

### Movement Classification

**Figure 7:**
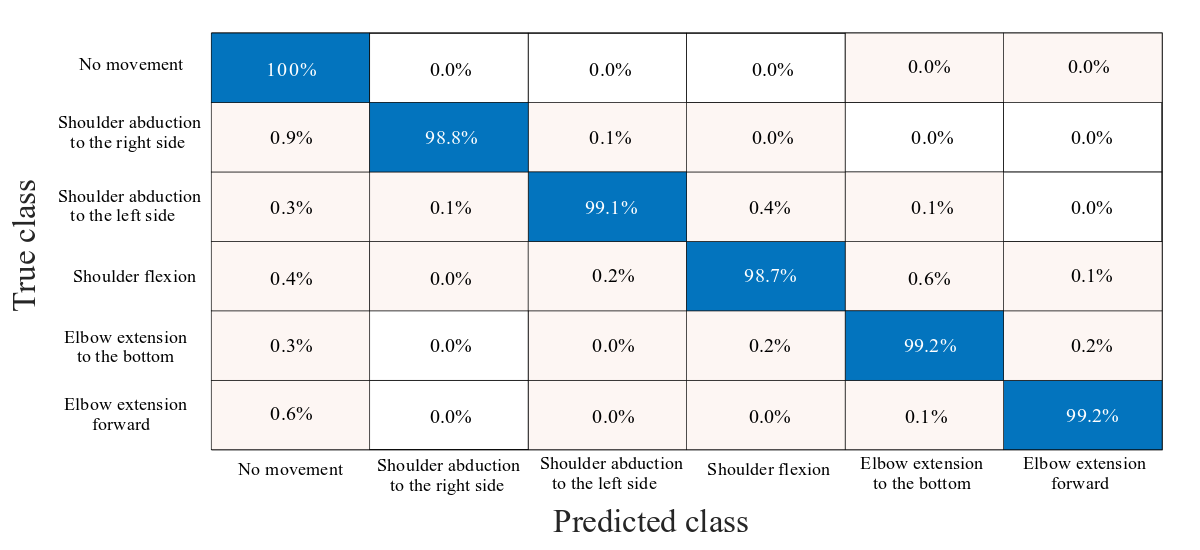
Confusion matrix summarizing the true positive rates of the classification algorithm. Blue entries denote instances of correct classification, whereas red entries denote instances of incorrect classification. The intensity of the color correlates with the true positive rate. Since the true positive (negative) rates for misclassification are very low, they appear in light pink.

Our classification model achieved an accuracy of 99.9%, where most misclassifications resulted from falsely classifying instances of movement as nonmovements (Figure 7). The true positive rate was highest for elbow extension to the bottom and for elbow extension forward, with 99.2% of instances classified successfully in both. The algorithm performed the worst in the classification of shoulder flexion forward, where the true positive rate reached 98.7%.

**Figure 8:**
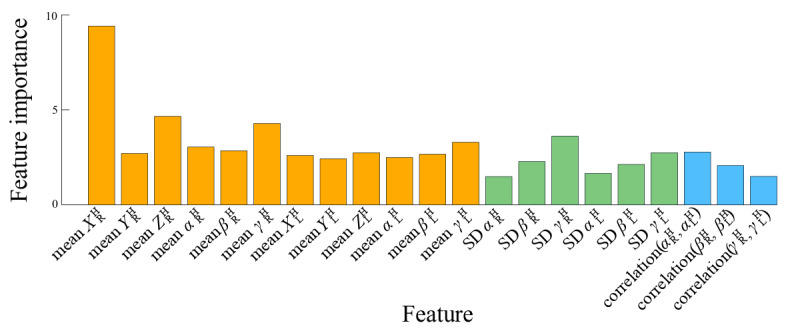
Feature importance based on out-of-bag error estimation, where importance is measured as the sum of decreases in error throughout all of the decision trees generated. Orange bars represent variable mean values, green bars represent their SDs, and blue bars represent the correlation of variable pairs.

Out-of-bag analysis revealed that the mean value of *X*^H^_R_ was the most important variable for the classification of movement, followed by the means of *Z*^H^_R_ and *β*^H^_R_ (Figure 8). The correlation between *α*^H^_R,_ and *α*^H^_L_ contributed the most to the classification among the correlation values. Among the SDs, *γ*^H^_R_ contributed the most to the classification. Nonetheless, correlation coefficients and SDs seemed to modestly impact the classification. The mean value of *γ*^H^_L_ was the least important, and *α*^H^_R_ had the smallest contribution to classification among the SD values.

## Discussion

### Principal Findings

As the world’s population is aging, the incidence of stroke and other neuromuscular diseases is increasing, and the demand for affordable and convenient physical therapy is rising [80]. Sensor and communication technologies are readily available for delivery and monitoring of home-based therapy; however, human interaction is a critical design aspect in this context: telerehabilitation programs are carried out without clinical supervision, so that patients must motivate themselves to perform exercises with sufficient intensity and frequency.

Lack of motivation has led to the study and development of exergames [17,81], where physical activity facilitates games. Although the effectiveness of these interventions has been demonstrated [81,82], it may be further maximized by incorporating cognitively challenging elements, learning, and sociality [83] as older adults, who comprise most patients, show a propensity toward these features [83]. As such, citizen science presents itself as an intellectually stimulating motivational framework with greater appeal to patients. By framing physical exercise in citizen science, patients would be able to learn about ongoing research, bring about scientific discoveries, and support a cause they care about—all while adhering to their rehabilitation regimen.

A second, yet equally important aspect in the design of telerehabilitation systems is minimizing health care providers’ time commitment such that they can diagnose and monitor multiple patients rapidly and simultaneously. However, this undertaking can become especially challenging when human behavior is abnormal [84]. Machine learning offers a viable means of automating the classification of human movements. Multiple examples exist where machine learning algorithms successfully detect and analyze different behaviors with high accuracy, as well as deviations from those behaviors, whether the application was for safe driving [85], gaming [86], or physical therapy [63-65]. Through machine learning algorithms, devices can learn from new data such that they can update their control strategies and dynamically adapt to the user’s behavior over time. This feature is particularly useful for telerehabilitation applications, as patients recover motor function and move differently [84,87].

In this study, we present the use of machine learning to identify and classify bimanual movements in VR. We demonstrate the approach in the context of a citizen science software that is dedicated for telerehabilitation. Commercial gaming systems are advantageous for home-based rehabilitation because they are relatively small, affordable, and user-friendly [88]. VR gaming systems are particularly favored as they confer high levels of immersion and increase user engagement [16,40,41,89]. In telerehabilitation, recovery is often hindered by patients’ lack of motivation to perform prescribed exercises [83]. Thus, the motivational aspects of home-based interventions are crucial to their success. To address this challenge, we also incorporated citizen science content into the application, such that the user could contribute to an authentic scientific project and help clean a polluted canal [32]. The task leverages human intellect as an intrinsic motivator and has a strong potential to improve patients’ sense of self-worth [32,88,90].

In all, 9 participants interacted with the citizen science system through a set of 5 predefined bimanual gestures. Bimanual training effectively improves rehabilitation outcomes through several physiological mechanisms [52,53,59]. This clinical approach could also target a wider range of patients with varying levels of impairment. Specifically, for the Oculus Rift system, a rigid link can be designed and 3D-printed for the Touch controllers such they are affixed to one another [91]. The custom-made link could enable passive exercise of the affected limb in patients with moderate to severe impairment, whereby the intact limb mediates coordinated movement of the paretic side. In a future study, we will seek to measure movements of participants with and without such fixture and compare its effect on motor performance.

One of the novelties of our approach lies in the application of a movement classification algorithm to a VR exercise for telerehabilitation. Although the movements we incorporated into game control are carried out along the 3 orthogonal anatomical planes and appear to be easily distinguishable, they require coordinated flexion or extension of the shoulder and elbow joints, as well as pronation of the forearms. For example, extending the right arm to the right side of the body involves simultaneous flexion of the shoulder, lateral rotation of the shoulder, extension of the elbow, and pronation of the forearm. Owing to these degrees of freedom, backward kinematics to determine the angles of these joints would require more information beyond the position of the Touch controllers relative to the head-mounted display. To further support this notion, our PCA results showed that the Tait-Bryan angles of the Touch controllers relative to the head-mounted display, and not their positions, are salient during movements. Most variations in these angles likely resulted from simultaneous movement of the shoulder and elbow joints and pronation of the forearm.

The variation of features based on relative angles is expected to become extremely important for the classification of movements when our approach is implemented on data from patients with stroke. Stroke can lead to a wide range of movement abnormalities, including spasticity, segmentation, and compensation. However, the latter is best known for sabotage rehabilitation efforts. In the face of reduced mobility, patients with stroke tend to recruit body parts that are not normally involved in certain movements to add degrees of freedom to their kinematics. For example, patients with stroke commonly use their trunk during reach movements to compensate for the limited range of motion of their upper limbs [92,93]. By reinforcing these strategies, patients perpetuate the nonuse of the affected limb and do not recover their function. Fortunately, compensatory movements would be easily detected through our algorithm, whereby the angles of the Touch controllers relative to the headset will not vary significantly.

The algorithm was used to classify the movements the participant performed toward a genuine telerehabilitation paradigm, where one’s motor performance is monitored remotely by a clinician. The algorithm classified bimanual movements objectively and reliably, reaching 99.9% accuracy. The 0.1% inaccuracy was mainly related to lack of sensitivity with respect to the presence of a movement. In other words, the algorithm erroneously classified movements as instances of no movement. This misclassification likely resulted from the use of a moving-window scheme. The moving window covers 13 time steps. During the algorithm training, the instantaneous true class of a window was defined as the mode of the true classes of the time steps it covered. For example, if the window covered 2 time steps of shoulder flexion and 11 time steps of no movement, its true class was *no movement*. At the beginning and end of each movement segment, the window covered 7 time steps of one class and 6 time steps of another class. The true class was then arbitrarily defined as 1 of the 2 classes. The accuracy of our approach may be further improved by refining this scheme and eliminating false negatives or by applying an alternative method to assign the true class of a moving window.

Future research could explore the use of alternative dimensionality reduction techniques. Our selection of features was based on the results of PCA, which informed us about which variables characterized each movement. However, this method may be inappropriate. In symmetrical movements performed by the participant, PCA showed that variables in only 1 arm were prominent. For example, when a participant performed shoulder abduction to the right side of the body, 2 angles of the left Touch controller and only 1 angle of the right Touch controller were dubiously deemed salient. Potentially, nonlinear dimensionality reduction methods such as Isomap, diffusion maps, and principal manifolds could better identify sets of variables that distinguish one movement from another [94-96].

The methodology presented herein can be extended to several research directions. First, multiple classification schemes can be applied in tandem to distinguish between static and dynamic poses. This will be especially useful for measuring metrics that are important for clinical evaluation, such as movement accuracy [97], smoothness [73,98], and coordination [99].

We measured some motor performance metrics using data collected by the VR system. We observed symmetry in motor performance when comparing the right and left arms. In patients with paresis, we expected significant differences in motor performance between each side of the body. Specifically, movements of the affected arm would present stiffness and be segmented early in recovery, measured through lower mean speed, reduced range of motion, and longer path lengths, which will change over time as muscle function is recovered in the affected arm. We also found considerable variation among healthy participants with respect to smoothness. It is tenable that this metric reflects the individualistic nature of user interaction with the VR interface, whether it involves abrupt initiation of movements or the sequential use of different sets of upper limb joints. As such, smoothness should be examined over the course of a movement rather than as a single score. To further support this notion, Rohrer et al [73] showed that the smoothness of pathological movements is characterized by a series of peaks and dips, which become shorter and shallower along recovery.

In addition to the quality of movements, one might consider the use of cognitive cues in the analysis to treat low motivation. Posture and movement have been previously demonstrated to be closely related to engagement [100,101]. For example, restlessness may be reflected by the frequently moving body weight between the legs. Similarly, arousal can be expressed by head rotation and extensive hand movements [102]. The combined use of biometrics, such as heart rate, skin conductance, and pupil dilation, may also provide important insights into human behavior [103-105]. Incorporating such psychophysiological sensory information could open the door for multifaceted interventions in telerehabilitation [106], although this path will require the use of additional sensors and requires further research.

Finally, the classification algorithm can be enhanced to detect and minimize compensatory movements. Compensatory movements are nonphysiological movements that patients with disabilities perform with their bodies to compensate for their limited range of motion. Essentially, the patients use muscles that are not normally involved in the movement, thereby adding degrees of freedom to it. Most commonly, patients tend to displace their torso during reaching tasks to compensate for their inability to move their upper limbs [92,107,108]. Although such nonphysiological movements improve patients’ function instantly, they are energetically inefficient, hinder functional recovery, and pose a risk of injury [109,110].

Recently, Cai et al [111,112] explored the effectiveness of machine learning in detecting compensatory movements in patients with stroke. In their experimental setting, users sat on a chair covered with a pressure distribution mattress and interacted with a tabletop robotic manipulator [112]. Data were collected on their motion from the mattress and from a VICON 3D motion capture system [112]. Users’ postures and compensation were classified by an SVM algorithm, which achieved an accuracy >96%. Although the sensors used in this study are different in nature from those of commercial VR gaming systems, the results are encouraging and suggest that our approach is feasible. Work to assess our approach is currently under way, and head-mounted display-based features are expected to aid in the detection of compensatory movements.

### Limitations

Our findings strongly support the viability of machine learning in the accurate assessment of movements in telerehabilitation with commercial VR systems. Nonetheless, the several limitations of this study must be acknowledged. First, this study was conducted on healthy participants only. Patients with stroke exhibit a wide range of movement disorders, including loss of mobility, loss of balance control, spasticity, chorea, and adoption of maladaptive movements [113-116]. It is unknown whether these disorders can be detected and correctly characterized from sensor data, let alone be tracked and monitored over time. We are currently collecting controlled clinical data from patients with stroke and intend to challenge these questions once the study is concluded.

The second limitation concerns the focus of our system on bimanual training with the Oculus Rift. Although this setting is practical, affordable, and has the potential to improve engagement in telerehabilitation, it is still subject to the limitations of machine-mediated patient–physician interactions. During in-clinic meetings, a physician can assess the physiological, behavioral, and emotional status of a patient simultaneously. For example, physicians may evaluate skin tactile feedback during grip [117] or the patient’s ability to balance while performing gross motor movements [118]. This cannot be accomplished in a telerehabilitation setting without teleconferencing with a physician or encumbering the patient with multiple wearable sensors, which would likely require special training and the aid of another person. Nonetheless, many of these in-clinic assessments may be feasible in telerehabilitation by means of machine learning. Emotion recognition from physiological [119,120] and behavioral [121,122] signals has already been demonstrated. Similarly, research has been carried out to predict patients’ ability to balance [123] and infer pain levels from kinematic features [124] and detect compensatory movements [125]. Thus, machine learning methodologies may successfully quantify other aspects of rehabilitation from data originating from a single modality, thereby providing health care providers with more information to monitor patients remotely.

Another nontrivial limitation of our study is the essence of machine learning as a black box [60,126-129]. In recent years, it has become widely accepted to trust machine learning predictions without fully understanding the model from which they are derived. The transparency of machine learning models is paramount to users’ trust in machines [60]. In medical applications, rather than perceiving decisions as arbitrarily made, an understanding of their rigor and potential sources of errors must be gained for good clinical decision-making. Furthermore, machine learning algorithms are vulnerable to adversarial attacks [127-129]. Minimal perturbations can significantly impact the output of algorithms and remain unnoticeable to human inspectors [127]. Thus, in future work, we will probe the model and apply perturbing strategies to interpret it [60].

### Conclusions

This study is a first step in our endeavor to incorporate machine learning into VR-mediated telerehabilitation. We classified bimanual movements using a bagged trees classifier and achieved high performance. Work to expand on our findings and hone our approach is underway, including experiments with patients with stroke, development of an interpretable model, and detection of compensatory movements.

## Abbreviations

HUD: heads-up display
PCA: principal component analysis
SVM: support vector machine
VR: virtual reality
